# Errors of Upright Perception in Patients With Vestibular Migraine

**DOI:** 10.3389/fneur.2018.00892

**Published:** 2018-10-30

**Authors:** Ariel Winnick, Shirin Sadeghpour, Jorge Otero-Millan, Tzu-Pu Chang, Amir Kheradmand

**Affiliations:** ^1^Department of Neurology, The Johns Hopkins University School of Medicine, Baltimore, MD, United States; ^2^Department of Neurology, Neuro-medical Scientific Center, Taichung Tzu Chi Hospital, Buddhist Tzu Chi Medical Foundation, Taichung, Taiwan; ^3^Department of Medicine, Tzu Chi University, Buddhist Tzu Chi Medical Foundation, Hualien, Taiwan; ^4^Department of Otolaryngology-Head and Neck Surgery, The Johns Hopkins University School of Medicine, Baltimore, MD, United States

**Keywords:** vestibular migraine, head tilt, subjective visual vertical, perception of upright, dizziness

## Abstract

Patients with vestibular migraine (VM) often report dizziness with changes in the head or body position. Such symptoms raise the possibility of dysfunction in neural mechanisms underlying spatial orientation in these patients. Here we addressed this issue by investigating the effect of static head tilts on errors of upright perception in a group of 27 VM patients in comparison with a group of 27 healthy controls. Perception of upright was measured in a dark room using a subjective visual vertical (SVV) paradigm at three head tilt positions (upright, ±20°). VM patients were also surveyed about the quality of their dizziness and spatial symptoms during daily activities. In the upright head position, SVV errors were within the normal range for VM patients and healthy controls (within 2° from true vertical). During the static head tilts of 20° to the right, VM patients showed larger SVV errors consistent with overestimation of the tilt magnitude (i.e., as if they felt further tilted toward the right side) (VM: −3.21° ± 0.93 vs. Control: 0.52° ± 0.70; *p* = 0.002). During the head tilt to the left, SVV errors in VM patients did not differ significantly from controls (VM: 0.77° ± 1.05 vs. Control: −0.04° ± 0.68; *p* = 0.52). There was no significant difference in SVV precision between the VM patients and healthy controls at any head tilt position. Consistent with the direction of the SVV errors in VM patients, they largely reported spatial symptoms toward the right side. These findings suggest an abnormal sensory integration for spatial orientation in vestibular migraine, related to daily dizziness in these patients.

## Introduction

Vestibular migraine (VM) is the most common cause of dizziness and spatial disorientation with a lifetime prevalence of about 1% in the general population (1). Currently the pathophysiology of vestibular migraine is unknown and a pathognomonic test is lacking (2). Although abnormal findings in vestibular evoked myogenic potentials (VEMP) imply disturbances of “low-level” otolithic pathways in these patients, the reduced motion detection thresholds in the roll plane and functional imaging data support the hypothesis that VM patients harbor a dysfunction of “high-level” vestibular perception (3–7).

A key aspect of our spatial perception is “orientation constancy”, as we maintain a stable perception of our surroundings in upright orientation despite continuous changes in the eye, head and body positions. Patients with vestibular migraine often complain of symptoms triggered by these changes, raising the possibility of dysfunction in neural mechanisms underlying orientation constancy (1). Such perceptual dysfunction can be studied by measuring perception of upright in a psychophysical task known as the subjective visual vertical (SVV) (8, 9). Perception of upright involves integration of graviceptive signals from the otoliths with visual inputs from the retina and proprioceptive inputs encoding the head, eye, and body positions (9–11). In the upright position, where the reference frames of the eye, head, and visual world are all aligned with the direction of gravity, SVV typically remains within 2° of earth vertical (9, 12). With lateral head tilts, however, there are usually systematic SVV errors that do not correspond with the magnitude of the head tilt (9). Naturally, a lateral head tilt leads to a change in the torsional eye position in the opposite direction of the head tilt. This ocular counter-roll only partially compensates for the amount of head tilt, typically with a low gain of about 0.10–0.25 in humans (13, 14). Therefore, the reference frames for the head, eye (retina) and the visual world no longer align with the gravitational vertical, and images become tilted on the retina during head tilt. This separation of the sensory reference frames introduces a challenge for the brain, especially in the absence of visual cues, when it has to rely on information about the head (in space) and eye (in head) positions to determine upright orientation. Such processing demand is reflected by the systematic SVV errors during head tilt (9). Usually, at small head tilt angles, SVV errors are in the opposite direction of the head tilt, reflecting overcompensation for the amount of tilt and thus overestimation of upright orientation relative to the head position (known as the E-effect) (9). At large tilt angles, SVV errors are usually in the direction of the head tilt, reflecting undercompensation for the amount of tilt and thus underestimation of upright orientation relative to the head position (known as the A-effect) (9, 15, 16).

Previous studies in patients with vestibular migraine found that SVV was not altered when the head was in the upright position (17, 18). However, in these patients, errors of upright perception have not been investigated during static head tilt, when the brain has to maintain a common multisensory reference frame for orientation constancy. Thus, here we asked whether such multisensory integration is affected in vestibular migraine by investigating the effects of static head tilts on SVV accuracy and precision, and comparing the results with those of healthy controls.

## Materials and methods

### Participants

We enrolled 54 participants: 27 healthy controls with no prior history of migraine, dizziness, or other neurological disorder, and 27 patients who met the diagnostic criteria for vestibular migraine according to the consensus document of the Bárány Society and the International Headache Society (IHS) (1). The experiments were approved by the Johns Hopkins institutional review board and informed written consent was obtained from all participants.

Patients were recruited consecutively from the Johns Hopkins Outpatient Center between March 2016 and June 2017. Control participants were also recruited within the same time period. The average age for healthy controls was 41 years old (16 female) and for patients was 43 years old (19 female). All participants were right-handed by self-report, except for one left-handed patient (34 y/o, female) and one left-handed control participant (34 y/o, female). All patients met the diagnosis of vestibular migraine based on the Bárány and IHS criteria (Table 1). Patients with peripheral or central vestibular dysfunction on exam or with lab or imaging findings that confirmed other diagnoses were not included in this study. Absence of vestibular dysfunction or central pathology was verified by expert neuro-otological examination, brain MRI, examination of the eye-movements using video oculography, video head impulse testing (vHIT), and quantitative rotational chair testing. None of the patients had spontaneous nystagmus with removal of visual fixation or provoked nystagmus with head shaking, vibration over the mastoids, hyperventilation, Valsalva maneuver, or in the static head down positions (i.e., positional/positioning nystagmus) to indicate an underlying vestibular imbalance (19). The ocular motor evaluations including saccade, pursuit, and optokinetic responses were normal. All patients had normal balance function that included evaluations with tandem gait, standing with heels together, and standing on one leg with eyes open and closed. Fourteen patients (51.9%) were not taking any CNS-acting medication. Four patients (14.8%) were on selective serotonin or serotonin and norepinephrine reuptake inhibitors (SSRI/SNRIs), four patients (14.8%) were on tricyclic antidepressants (TCAs), and one patient (3.7%) was on trazodone. Two patients (7.4%) were taking valproic acid, one patient (3.7%) carbamazepine, and one patient (3.7%) topiramate. Four patients (14.8%) were on meclizine and three patients (11.1%) were on benzodiazepines. A dizziness questionnaire was used to probe the quality of spatial symptoms in VM patients. Specifically, patients were asked about sensation of body tilting or pulling, sensation of body rotation or spinning, dizziness when lying down on the sides, or dizziness with tilting the head laterally to the shoulders. If any of these qualities was present, they were asked to specify the direction in which they experienced symptoms as rightward, leftward, rightward and leftward, or other directions. All patients reported daily dizziness with a mean duration of 2 years (range: 3 months to 12 years, standard error of the mean: 6 months).

**Table 1:** Symptoms characteristics and vestibular test results in VM patients.

**Characteristics of dizziness**		**Vestibular tests results**	
	***n* (%)**	***vHIT***	**Mean (SEM)**
Moderate intensity	6 (22.2)	Gain, left	0.96 (0.02)
Severe intensity	21 (77.8)	Gain, right	1.00 (0.02)
Lasting minutes	2 (7.4)	vHIT gain asymmetry	0.04 (0.01)
Lasting hours	25 (92.6)		
		***Chair rotation velocity steps***	**Mean (SEM)**
**Characteristics of headaches**		60°/s gain, left	0.64 (0.03)
		60°/s gain, right	0.69 (0.04)
	**Mean (SEM)**	240°/s gain, left	0.57 (0.04)
Age of onset (years)	30 (3.5)	240°/s gain, right	0.59 (0.03)
Frequency (days per month)	6.5 (1.8)	60°/s TC, left	18.09 (1.30)
Intensity (1 to 10)	5.7 (0.5)	60°/s TC, right	17.45 (1.30)
	***n*** **(%)**	240°/s TC, left	12.82 (1.00)
Lasts 4 h or more	14 (60.8)	240°/s TC, right	12.99 (0.77)
Unilateral	12(77.3)	60°/s gain asymmetry	0.12 (0.01)
Pulsatile or throbbing	16 (61.5)	240°/s gain asymmetry	0.07 (0.02)
Aggravation by physical activity	7 (33.3)	60°/s TC asymmetry	0.15 (0.02)
Visual aura	11 (45.8)	240°/s TC asymmetry	0.07 (0.02)
Photophobia	19 (79.2)		
Phonophobia	19 (79.2)		
Nausea and/or vomiting	7 (29.2)		

*All patients had chronic daily dizziness and met the diagnostic criteria for vestibular migraine. The video head impulse testing (vHIT) results show normal vestibular gains (eye velocity/ head velocity) with both right and left head impulses (normal gain: 0.7–1) (20). The rotational chair results show normal vestibular gains (normal gain: 0.4–1) at both low and high velocity steps (60°/s & 240°/s). The time constant (TC), which is a measure of nystagmus duration induced by the rotational velocity step, is also within the normal range at both velocities (normal range: 8–25 s) (21). Overall, there are no significant asymmetries between the right and left vestibular gains (i.e., both vHIT and rotational chair testing) or time constants. n, Number of patients; SEM, Standard error of the mean*.

### Experimental setup for SVV recordings

Participants sat upright in a completely lightproof room, fixing on a red dot (diameter 1.67 mm) at eye level, which was presented on an active matrix LED screen (2,560 × 1,600 AMOLED, Samsung Galaxy Tab S) 55 cm away in front of them. We chose this type of tablet because its pixels are not backlit, eliminating any glow from the black screen background that might provide visual cues during SVV recording. In addition, subjects could only see the screen through a round opening, as the frame of the tablet mount was also covered by gaffer's tape to avoid reflections. Participants wore contact lenses or glasses as needed. SVV was measured in three head positions for each participant: upright (UP), 20° head tilt toward the right shoulder (right ear down or RED), and 20° head tilt toward the left shoulder (left ear down or LED). A molded bite-bar secured to a rotating tilt plate was used to passively position the head in the roll plane, and for measuring the angle of head tilt. Each participant was tested under all three head tilt positions in random succession, completing 100 SVV trials in each head position. We chose 20° head tilt because it is within the physiologic range of neck positions, and while comfortable to maintain during the recordings, it is large enough to induce SVV errors.

### SVV paradigm

**Figure 1:**
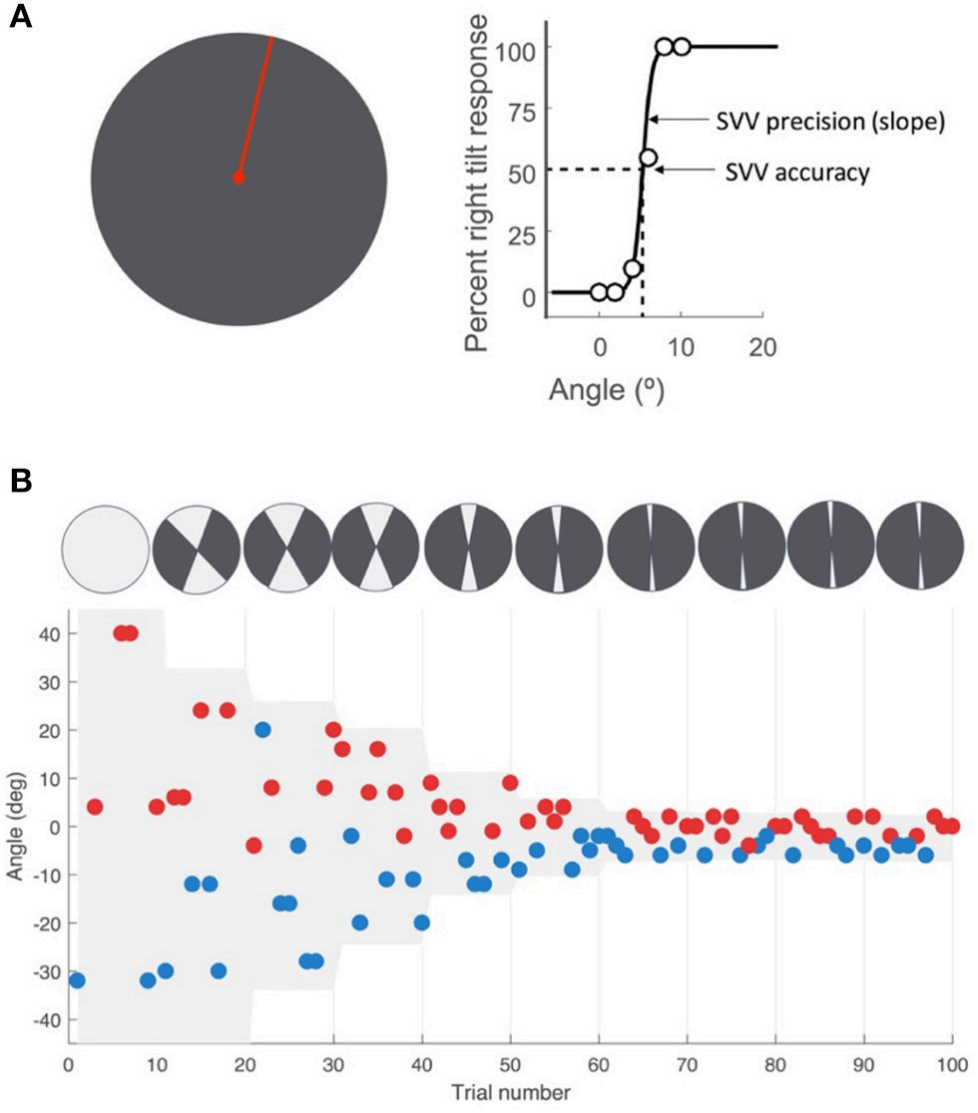
SVV paradigm. **(A)** SV V measurement with the line stimulus (red) presented at a random orientation in each trial. As a two-alternative forced choice paradigm (2AFC), the task in each trial is to report whether the line is tilted to the right or left of perceived upright orientation. SV V is then determined by fitting a psychometric curve to the responses from all trials, and calculated as the value on the curve at which the probability of left or right responses is 50% (point of subjective equality). The SV V precision is calculated as the slope of the psychometric fit. **(B)** A sample time course of 100 trials with the participant's responses, with each point representing one trial. The y-axis shows the angle of the line presented and the color indicates the response for that trial. The left tilt responses are shown in blue and the right tilt responses in red. The line angles were presented randomly within a range that started at 360° and then adjusted based on previous responses (illustrated by the top circles with the light gray sectors). At the end of every 10 trials, the center of this range (also shown in light gray shade on the graph) was set as the SV V value calculated from the previous trials. The range was reduced in half every 10 trials until it reached 8° (±4° around the calculated center), after which it was kept constant for the remaining trials.

We used a two-alternative forced choice task (2AFC) that was not bound by fixed probing angles to measure SVV responses. The full description of this SVV paradigm has been previously published by our group (22). At each trial, a red line (length 4 cm, width 0.75 mm) was presented at a random angle, radiating from a red dot (Figure 1A). The paradigm was controlled by a custom software written in Matlab (Mathworks) using Psychtoolbox (23). Stimuli were transmitted from the Matlab computer to the display tablet over the network using join.me software (LogMeIn, Inc.). In each trial, participants clicked one of two buttons on a game controller, reporting whether the line was oriented to the “right” or “left” of their perceived vertical orientation. The paradigm started with angles presented randomly from the entire 360° range. As the recording session progressed, the range of probing angles was adjusted in blocks of 10 trials by centering it around the SVV calculated from responses in previous trials (Figure 1B). Each block consisted of five different angle orientations in the upper visual field, and five in the lower visual field. After the sixth block, the range was kept constant at 8°. If a trial was missed when the participant did not respond within 1.5 s, that angle was presented again at a later time within the same block, ensuring that all angles were probed and the corresponding responses were obtained only once. Upon completion of 100 trials (3–5 min), an SVV value was calculated by fitting a psychometric curve to the responses from 100 trials (Figure 1A). The angle at which the probabilities of left and right responses were both 50%, the point of subjective equality, was taken as the SVV value. An estimate of the slope of the psychometric curve was used to calculate SVV precision. This was calculated as the difference in angle between the two points on the psychometric curve with probabilities of 50% and 75%.

### Data analysis

SVV accuracy in each head tilt position was compared between the VM patients and healthy controls using unpaired *t*-tests with the α-level adjusted to 0.0167 by Bonferroni correction. We used D'Agostino-Pearson omnibus test to verify the normal distribution of the SVV results. The results for SVV precision in each head tilt condition were compared similarly between the VM patients and healthy controls.

## Results

**Figure 2:**
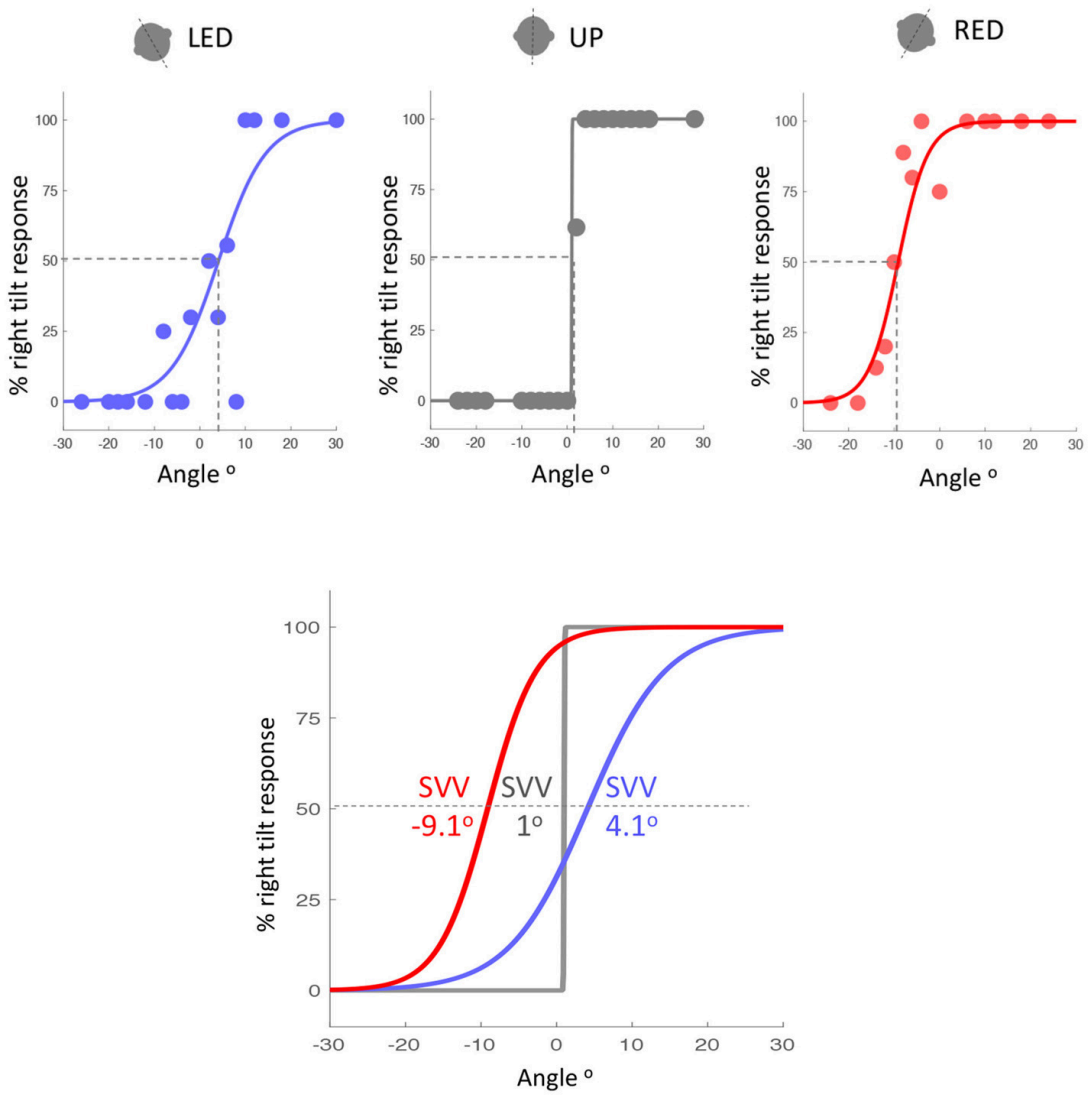
Example of SVV accuracy in a VM patient during the left tilt (blue), upright (gray) and right tilt (red) head positions (top graphs). SVV is the point on the psychometric curves at which the probability of left or right responses is 50% (dashed lines). The psychometric curves and SVV values for the three head tilt positions are also shown together (bottom graph). Positive values indicate SVV errors towards the right side, and negative values indicate SVV errors towards the left side. SVV error is in the opposite direction of the head tilt (blue and red curves) and it is larger during the right head tilt position. LED, left ear down; UP, upright; RED, right ear down.

**Figure 3:**
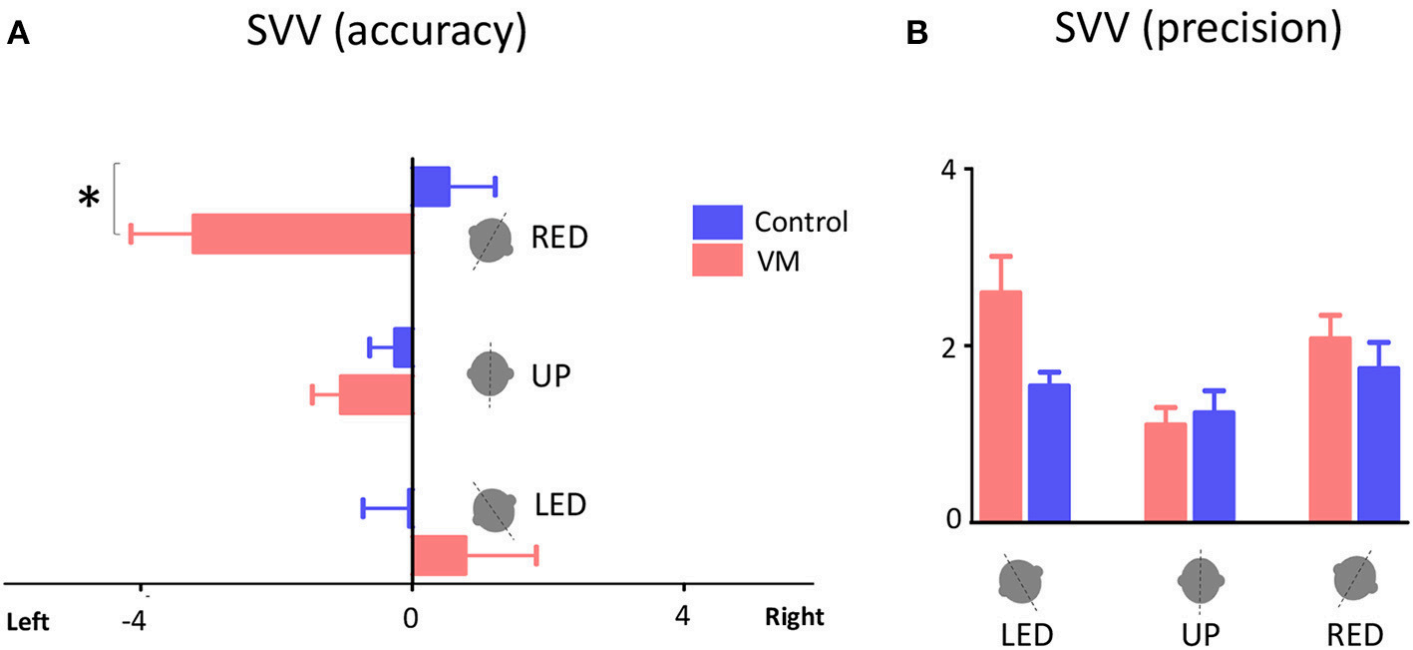
SVV accuracy and precision. **(A)** Mean values of SVV accuracy for VM patients and healthy controls with error bars showing standard errors of the mean (SEM). Positive values indicate SVV errors toward the right side, and negative values indicate SVV errors to the left side. The asterisk indicates a significant difference in SVV errors during right head tilt in VM patients compared to controls (*p* = 0.002). **(B)** Mean values of SVV precision with error bars showing SEM. LED, left ear down; UP, upright, RED, right ear down.

Accuracy of SVV responses (i.e., SVV error) in the three static head tilt positions (i.e., UP at 0°, left head tilt at −20°, and right head tilt at +20°) were compared between VM patients and healthy controls. The mean SVV error in the upright position was within the normal range (within ±2° of earth vertical) for both VM patients (mean SVV± SEM: −1.04° ± 0.43) and controls (−0.25° ± 0.38) (9, 12), and it did not differ between the two groups (Student's *t*-test with Bonferroni correction α = 0.0167; *p* = 0.17). With the left head tilt, the mean SVV error in VM patients (0.77° ± 1.05) and controls (−0.04° ± 0.68) were not different (*p* = 0.52). With the right head tilt, the SVV error in VM patients (−3.21° ± 0.93) and controls (0.52° ± 0.70) were significantly different (Student's *t*-test with Bonferroni correction α = 0.0167; *p* = 0.002) (Figures 2, 3). Despite the difference in the SVV accuracy, the precision of SVV responses did not differ significantly between the VM patients and controls in any head tilt position (Student's *t*-test with Bonferroni correction α = 0.0167; *p* > 0.3 for all three head positions) (Figure 3).

We also analyzed the number of participants in each group that showed SVV errors with “overestimation” of the head tilt (SVV error in the opposite direction of the head tilt, i.e., the E-effect) or “underestimation” of the head tilt (SVV errors in the same direction as the head tilt, i.e., the A-effect) (Table 2). Participants whose SVV value was 0° for a given head tilt were classified as having neither the A- nor the E-effect (only two participants among controls). For VM patients, there were 14 A-effects (mean SVV ± SEM: −3.41° ± 0.73) and 13 E-effects (5.28° ± 1.04) with the left head tilt, while there were seven A-effects (2.43° ± 0.52) and 20 E-effects with the right head tilt (−5.19° ± 0.88). For controls, there were 14 A-effects (−2.75° ± 0.52) and 11 E-effects (3.40° ± 0.65) with the left head tilt and 14 A-effects (3.34° ± 0.60) and 13 E-effects (−2.51° ± 0.54) with the right head tilt.

**Table 2:** Number of A- and E-effects for the left (LED) and right (RED) head tilt positions.

	**Controls**		**VM patients**	
	**A-effect**	**E-effect**	**A-effect**	**E-effect**
LED	14	11(2^*^)	14	13
RED	14	13	7	20

*The asterisk indicates participants whose SVV value was 0° for a given head tilt, classified as having neither A- nor E-effect. Head positions: LED, left ear down; RED, right ear down*.

Overall, 21 VM patients reported dizziness induced by lateral body or head tilt or had sensations of body tilting, pulling, or rotation (Table 3). From these 21 patients, 16 (~75%) reported rightward symptoms, one reported leftward symptoms, one reported both rightward and leftward symptoms, and three reported symptoms in other directions. Six other patients that did not report these symptoms had unsteadiness mainly from a sense of motion of the environment. Thus, similar to the SVV errors, there was an asymmetry in spatial symptoms reported by VM patients.

**Table 3:** From all VM patients who reported spatial symptoms as sensations of body tilting, body pulling, body rotation, or dizziness with lateral body or head tilt, 16 patients (~75%) had rightward symptoms.

**VM spatial symptoms**			
Rightward 16	Leftward 1	Rightward & leftward 1	Other directions 3

## Discussion

Patients with vestibular migraine often experience dizziness and disorientation with changes in the head and body positions. Such symptoms raise the possibility of dysfunction in neural mechanisms that subserve spatial orientation. Here we investigated SVV errors during head tilt in patients who met the diagnostic criteria for vestibular migraine. When the head is tilted, the brain has to integrate sensory information that encodes the positions of the eye, head and body in order to maintain perception of upright. Our results show that SVV accuracy in VM patients was significantly worse during the right head tilt position. The larger SVV errors in VM patients were in the opposite direction of the head tilt position, consistent with overestimation of the tilt magnitude in the process of perceiving upright orientation. There was no difference in SVV precision between the VM and control groups at any head position, showing that the poor accuracy (i.e., larger SVV errors) in VM patients cannot be related to the variability of responses across SVV trials. VM patients had no signs of vestibular or ocular motor dysfunction that could lead to abnormal SVV deviations. On this basis, SVV deviations in these patients could be linked to a “higher order” dysfunction in multisensory integration for spatial orientation (i.e., vestibular and somatosensory inputs that encode head, neck and eye positions). Such a mechanism is in line with the potential role of multisensory integration in migraine pathophysiology (24). Consistent with the larger SVV errors during the right head tilt, the majority of VM patients reported spatial symptoms towards the right side, suggesting a link between the symptoms and SVV bias in these patients.

Previous studies have reported no difference in SVV errors in VM patients compared to healthy controls, although there was a higher variability in VM patients (25). These measurements were only made in the upright position, even though VM patients typically complain that symptoms are triggered or worsened with changes in the head or body position. Our results show similar SVV errors in VM patients and healthy controls with the head in upright position. However, there were larger SVV errors in VM patients during head tilt, in agreement with previously-reported reduced tilt perception thresholds (i.e., motion in the roll plane) in these patients (26, 27). These findings together suggest that VM patients may be sensitive to displacements in the roll plane, and their overestimation of the tilt position may lead to larger errors of upright perception.

The asymmetric effect of head tilt on upright perception in VM patients does not conform to the known perceptual biases seen in healthy individuals, which generally do not exhibit significant asymmetries between equal head tilts in both directions (9, 22, 28). Normally, with head tilts of less than 60°, healthy individuals show SVV biases, consistent with either the A-effect or the E-effect (9, 12). Here, our patients showed significantly larger E-effect during right head tilt (i.e., tilt overcompensation error). This asymmetry in SVV errors was consistent with the direction of spatial symptoms, which was also mainly to the right side. These findings show a plausible link between the SVV bias and dizziness in these patients. With no vestibular or ocular motor dysfunction, the errors of upright perception in VM patients could be linked to neural processes within the cerebral hemispheres that contribute to spatial orientation (9). In this context, a functional laterality has been shown in vestibular processing, postural control, perception of self-motion and spatial orientation (29–33). Likewise, the asymmetry in spatial symptoms and—consistent with that—errors of upright perception in VM patients might be related to distinct abnormalities in hemispheric interactions in processing sensory information for spatial orientation (e.g., vestibular or somatosensory inputs). Currently, little is known about these multisensory neural processes and they need to be addressed in future studies. Another possibility to consider is that VM pathophysiology might involve the vestibulo-cerebellum (i.e., nodulus/uvula), where vestibular inputs are processed with respect to their underlying rotational, gravitational, and translational components (34–36). A vestibulo-cerebellar dysfunction can affect perception of head tilt position and thus result in SVV deviation (34, 35, 37, 38). In our patients, however, we did not find any clinical signs of vestibulo-cerebellar dysfunction; e.g., ataxia, head shaking induced nystagmus or abnormality in the time constant of vestibulo-ocular responses with rotational chair testing. In this study, we did not measure torsional eye position along with SVV responses. Thus, even though we did not find clinical signs of vestibular imbalance in our VM patients, we cannot entirely rule out the possibility of SVV deviations from asymmetrical changes in ocular torsion during head tilt (i.e., otolith-ocular imbalance). This is, however, less likely as the SVV errors in VM patients were larger that it could be attributed to abnormality in ocular torsion alone. Future studies will have to address this issue, using simultaneous ocular torsion and SVV measurements during head tilt. Moreover, in order to parse out sensory contributions to spatial misperception in VM patients, SVV errors should be interpreted with respect to measurements of head tilt perception using a wider range of head tilt positions.

In conclusion, here we investigated orientation constancy in patients with vestibular migraine by measuring errors of upright perception during static head tilts. Patients with vestibular migraine, compared to healthy participants, showed larger errors of upright perception that were asymmetrical and were present primarily in one head tilt direction. Consistent with these perceptual errors, VM patients reported spatial symptoms towards the same direction. These findings, in the presence of normal vestibular function, suggest an abnormal sensory processing and integration for spatial perception in patients with vestibular migraine.

## Ethics statement

This study was carried out in accordance with the recommendations of Johns Hopkins Institutional Review Board (IRB). All subjects gave written informed consent in accordance with the Declaration of Helsinki. The protocol was approved by the Johns Hopkins IRB.

## Author contributions

AW, JO-M, SS, and AK contributed to the design of the study, running the experiments, analyzing the data, and writing the manuscript. T-PC contributed to analyzing the data and writing the manuscript.

### Conflict of interest statement

The authors declare that the research was conducted in the absence of any commercial or financial relationships that could be construed as a potential conflict of interest.
